# Editorial: Mental health issues in Southeast Asia regions: looking back and moving forward

**DOI:** 10.3389/fpsyt.2023.1229079

**Published:** 2023-06-09

**Authors:** Kit-Aun Tan, Shian-Ling Keng, Mansor Abu Talib

**Affiliations:** ^1^Department of Psychiatry, Faculty of Medicine and Health Sciences, Universiti Putra Malaysia, Selangor, Malaysia; ^2^Department of Psychology, Jeffrey Cheah School of Medicine and Health Sciences, Monash University Malaysia, Selangor, Malaysia; ^3^Centre of Research and Mental Health and Wellbeing, UCSI University, Kuala Lumpur, Malaysia

**Keywords:** mental health, Southeast Asia, prevention, assessment, evaluation, management

Mental health has not been given high priority in Southeast Asian countries. Looking back, in some Southeast Asian countries, the healthcare sector has primarily focused on infectious and tropical diseases, and other emerging public health concerns. Mental health, which falls under the domain of non-communicable diseases, has received comparatively less attention.

Southeast Asian nations differ in terms of their economic progress and per capita income, with some countries being more developed and prosperous, while others face greater disparities and lower level of income. These differences contribute to variations in the availability of resources and services related to mental health.

Despite the fact that Southeast Asia comprises ~8.58% of the global population, authors from this region have not received proportional attention or inclusion in the body of literature addressing mental health. This gap in representation can impede the advancement of holistic and culturally appropriate approaches to mental health interventions and services within the region.

In response, the 12 articles composing this unique *Frontiers Research Topic* seek to provide a clearer understanding of how mental health issues can be prevented, assessed, evaluated, and managed. Of these articles, most were quantitative, only one was qualitative, and one combined quantitative and qualitative. It is notable that most of the quantitative studies were cross-sectional by design. There is also one review article. We organized the articles in this Research Topic following the themes of prevention (three studies), assessment (three studies), evaluation (five studies), and management (one study) to give readers a cohesive structure and easier navigation (see Figure 1).

**Figure 1:**
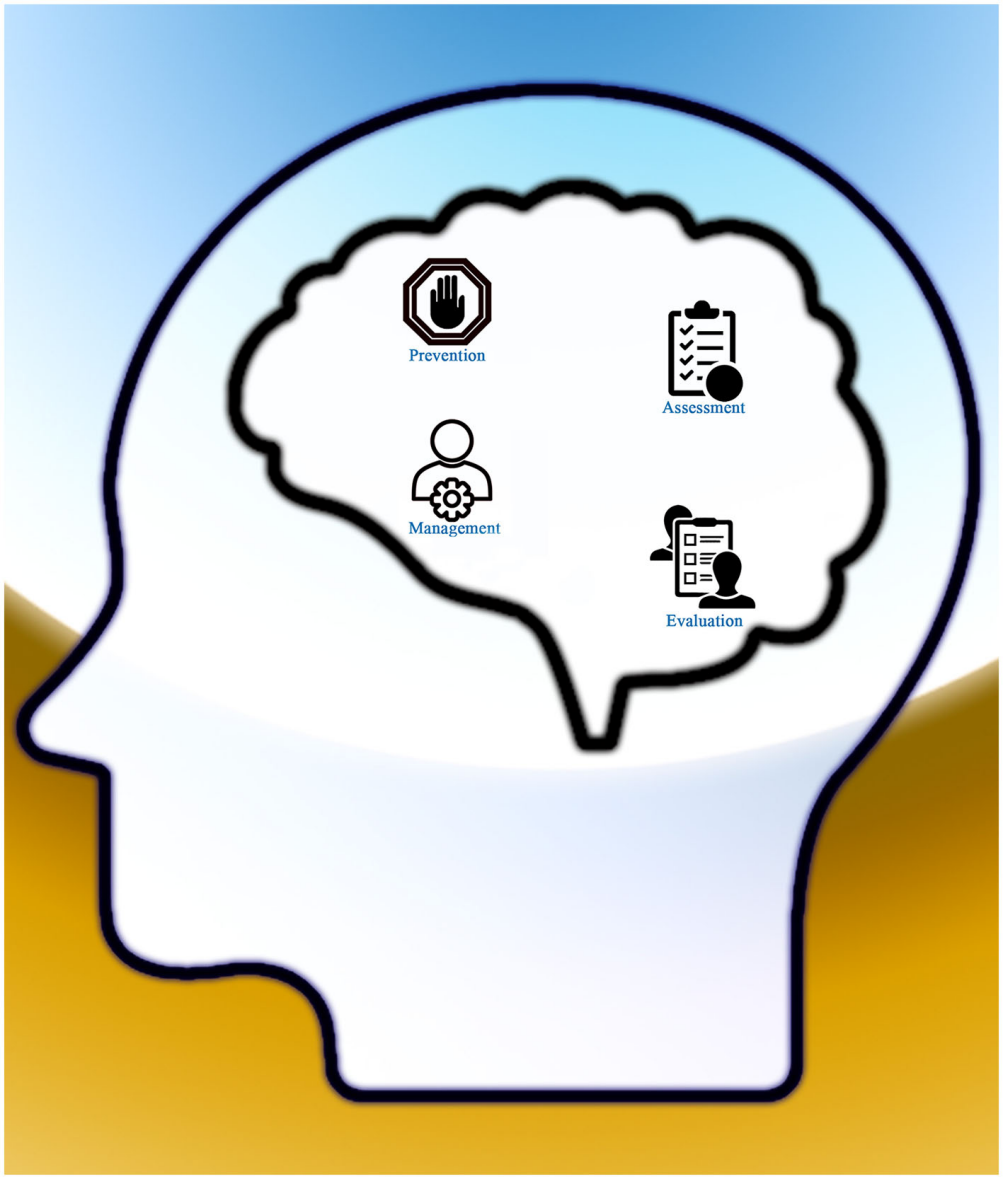
An integrative depiction of themes covered in this Research Topic.

Focusing on the theme of *prevention*, Abd Hadi et al. conducted a qualitative formative analysis that extended the conceptualization of social and emotional competencies in a local context. This expansion was achieved by integrating Asian cultural values such as preservation of interpersonal relationships. The study suggests a potential treatment strategy for emotional regulation that is culturally sensitive to Malaysian adolescents. In a single-arm prospective study, Roslan et al. reported the development, as well as examination of an online advanced suicide prevention gatekeeper training program aimed at raising awareness and self-efficacy in dealing with suicidal individuals in healthcare educators. The potential exists to expand this program to a wider population of healthcare educators in other Southeast Asian settings. Such expansion would involve providing these healthcare providers with hands-on skills to act as gatekeepers. A mixed-method study by Ke et al. reported that exposure to coastal environments and stress reduction were associated with psychological wellbeing in coastal communities from Malaysia and Indonesia.

Focusing on the theme of *assessment*, Adnan and Matore developed an index measuring adversity quotient—the capability to effectively handle challenges and convert obstacles into favorable circumstances—for use in a sample of pre-service teachers during practicum training. Investigating the psychometric properties of the Malay Version of the Beck Anxiety Inventory (Malay-BAI), Ismail et al. found that the Malay-BAI is a reliable and valid tool for assessing anxiety in a sample of adolescents in Malaysia. Efforts to validate psychological scales were shown in another study by Amin et al. The authors revealed that the Indonesian Version of the Scale for the Assessment of Negative Symptoms, a measure of negative symptoms in patients with schizophrenia, has robust psychometric evidence that supports its use in local mental health setting.

Focusing on the theme of *evaluation*, Ibrahim et al. found that those who were younger, had lower physical activity levels, and engaged in problematic internet usage demonstrated more severe symptoms of depression in a sample of Malaysian adolescents. In two studies involving university students, Sahimi et al. reported that excessive smartphone use was linked to high social anxiety and low self-esteem and quality of life, whereas Ooi et al. reported that those who experienced lower depression and anxiety reported higher life satisfaction when perceived burdensomeness (i.e., as a moderator) was low and that those who experienced lower stress reported higher satisfaction with life when thwarted belongingness (i.e., as a moderator) was high. As far as clinical population is concerned, the findings of Wahad et al. concluded that childhood emotional and physical abuse were negatively correlated to parenting satisfaction in a sample of women abusing amphetamine-type stimulant. The positive correlations between job stress and depression, as reported by Nordin et al., were stronger among anti-drug professionals who exhibited higher levels of avoidant coping (i.e., as a moderator) or lower levels of control coping (i.e., as a moderator).

Focusing on the theme of *management*, Kamaruddin et al. in their systematic review and meta-analysis suggested that the dynamic of social-online interactions is central in promoting cyber awareness and media literacy in existing classroom instructions. The authors called for the engagement of all stakeholders, particularly field-level practitioners, for developing effective interventions with emphasis on identification, prioritization, and planning.

## Conclusion

In conclusion, while momentum toward greater inclusion of Southeast Asian authors in mental health literature grows, this Research Topic represents just a small step in expanding Southeast Asia-based research representation. Notably, the studies in this article collection mainly stemmed from Malaysia and Indonesia. Moving forward, we strongly advocate for the inclusion of culturally diverse studies conducted in various regions within Southeast Asia. This line of studies will bring valuable insights and perspectives vis-à-vis prevention, assessment, evaluation, and management of mental health issues within the region.

## Author contributions

K-AT contributed to the conception of the Research Topic, wrote the first draft of the editorial, and revised the editorial for intellectual content. S-LK reviewed and edited the editorial. All authors approved the submitted version.

